# The bench-top accuracy of the VerteTrack spinal stiffness assessment device

**DOI:** 10.1186/s12998-020-00331-8

**Published:** 2020-08-18

**Authors:** Anika Young, Michael S. Swain, Gregory N. Kawchuk, Arnold Y. L. Wong, Aron S. Downie

**Affiliations:** 1 Department of Chiropractic, Faculty of Science and Engineering, Macquarie University, Sydney, Australia; 2 Department of Physical Therapy, Faculty of Rehabilitation Medicine, University of Alberta, Edmonton, Canada; 3 Department of Rehabilitation Sciences, The Hong Kong Polytechnic University, Hung Hom, Hong Kong

**Keywords:** Spinal stiffness, Spinal stiffness assessment, Mechanical spinal stiffness device, VerteTrack and instrumented spinal stiffness measurements

## Abstract

**Background:**

The assessment of spinal stiffness by manual palpation in clinical settings has demonstrated both poor accuracy and reliability. More recently, mechanical methods for assessment of spinal stiffness have demonstrated superior accuracy and reliability. However, mechanical methods of spinal stiffness assessment can be expensive, time consuming and/or unsuited to clinical practice. While a new device has been designed to address these issues (VerteTrack), its benchtop performance remains unknown.

**Aim:**

To measure the bench-top performance of VerteTrack.

**Methods:**

A series of laboratory-based experiments were conducted in February 2018 to investigate the accuracy (precision and bias) of load and displacement measurements obtained by VerteTrack and then were compared against an appropriate reference standard. Measurements of both multiple-level continuous assessment (multiple spinal levels measured), and single-level assessment (single spinal level measured) were performed on a viscoelastic foam medium (AIREX® balance beam, Switzerland) and the resulting stiffness calculated.

**Results:**

VerteTrack demonstrated high precision at all loads and displacements. There was minimal systematic measurement bias identified for applied versus reference load (mean bias = − 0.123 N; 95%CI − 0.182 to 0.428 N, *p* < .001), and no systematic measurement bias for measured versus reference displacement (mean difference = 0.02 mm; 95%CI − 0.09 to 0.14 mm, *p* < .001). The magnitude of stiffness obtained during multiple-level continuous assessment was on average 0.25 N/mm (2.79%) less than that for single-level assessment (95%CI − 0.67 to 0.17 N/mm, *p* < .001).

**Conclusions:**

VerteTrack demonstrated high accuracy (high precision, low bias) under bench-top conditions. The difference in stiffness found between multiple versus single spinal levels should be considered in the research context, but is unlikely to be clinically relevant. The results of this study demonstrate that VerteTrack may be suitable for both single and multi-level spinal stiffness measurements in-vivo.

## Introduction

**Fig. 1:**
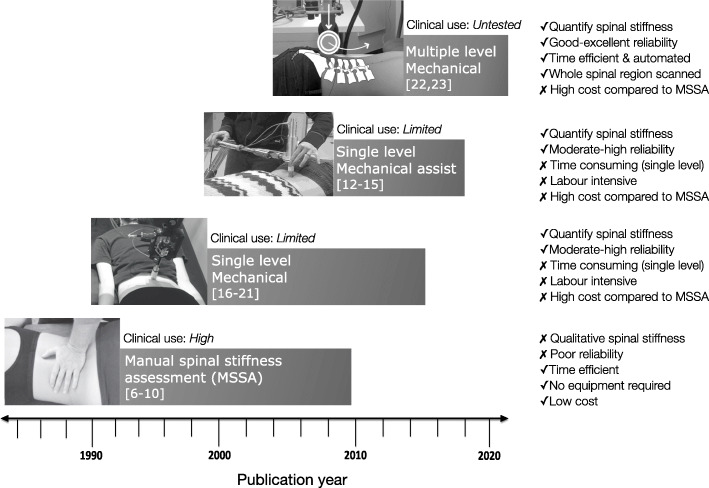
The evolution of spinal stiffness assessment

Manual therapists’ commonly use manual spinal stiffness assessment (MSSA) to guide diagnosis and treatment decisions for patients with non-specific spinal pain [1, 2]. In MSSA, therapists apply a force to the spine by hand in a posterior to anterior direction then subjectively interpret the resultant resistance to displacement [3]. Although MSSA is an accessible option for clinical practice, the reliability and criterion validity of this method is poor [3–10]. Mechanical devices were created as an objective alternative to MSSA [11, 12]. Fig. 1 illustrates the evolution of spinal stiffness assessment devices that typically assess spinal stiffness at one segmental level per indentation (single-level mechanical assessment) [12–21] which can be time-consuming and requires large amounts of data transcription.

**Fig. 2:**
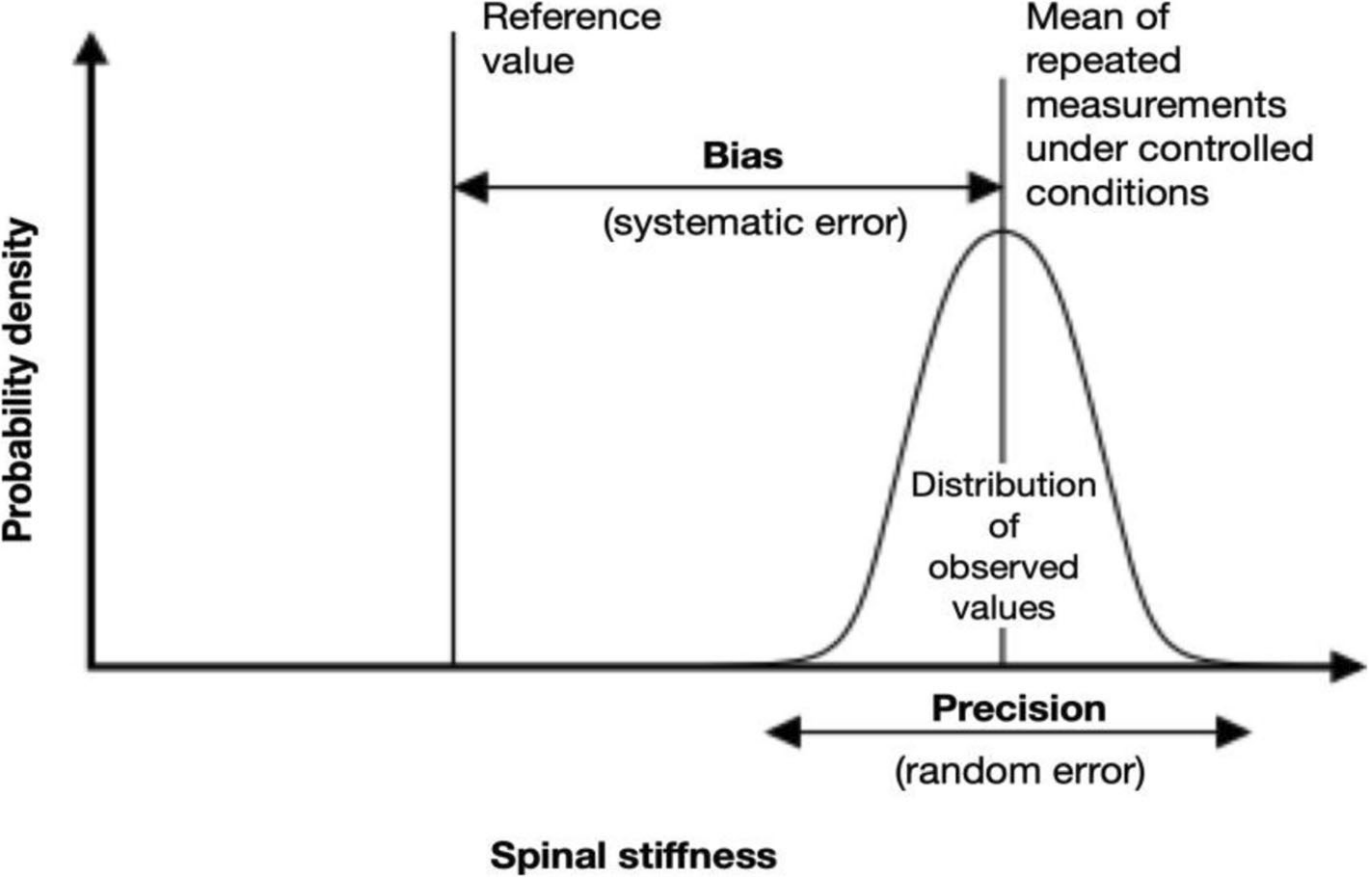
Components of measurement accuracy

VerteTrack is a new device developed to address the limitations of existing mechanical devices that quantifies bulk measurement of spinal stiffness [22]. A novel feature of the VerteTrack is a pair of rolling indentation wheels that enables stiffness assessment of an entire spinal region thereby minimising assessment time [22]. The VerteTrack has recently demonstrated excellent within-session test-retest reliability (intraclass correlation coefficient ICC_3, k_ 0.95–100) and good to excellent between-day reliability (ICC_3, k_ 0.82–0.93) in the clinical setting [23], however, its accuracy is unknown. Benchtop performance of a test instrument can be evaluated through measurement of precision (random error) and bias (systematic error) of the system under test (Fig. 2) [24, 25]. Therefore, this study aimed to measure the bench-top performance of the VerteTrack under both single-level and multiple-level continuous test conditions.

## Methods

### Study design, setting and equipment

This was a laboratory-based accuracy study conducted in February 2018 [24]. Experiments were designed and conducted following the International Organization for Standardization (ISO 5725-1) for the accuracy of measurement methods and results [24].

### VerteTrack overview

**Fig. 3:**
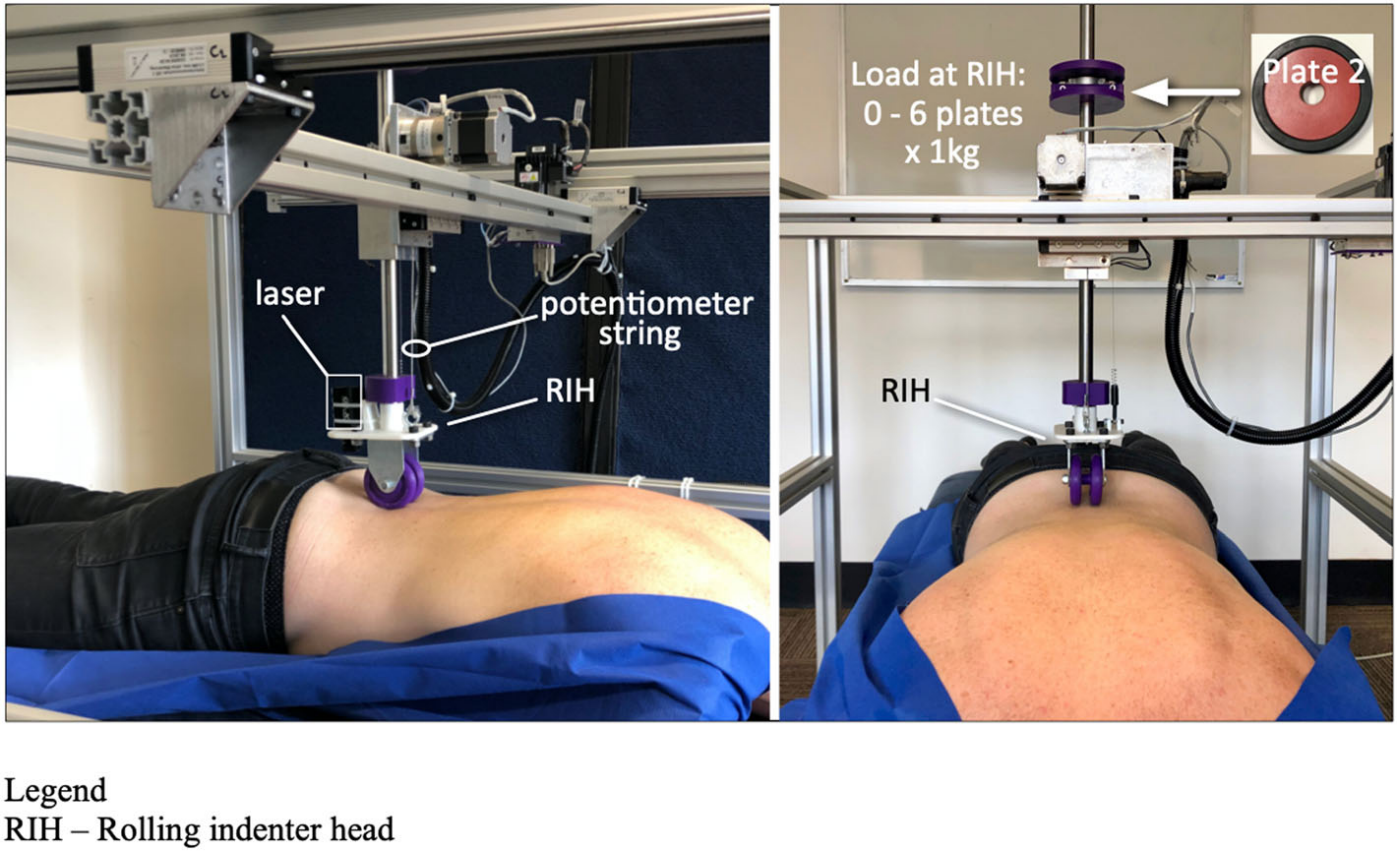
A labelled image of the VerteTrack during indentation]. Legend: RIH – Rolling indenter head

The VerteTrack frame (width 1080 mm × height 1090 mm × length 1, 510 mm) suspends an aluminium gantry that supports movement of a rolling indenter head (RIH) in three axes: X-axis (longitudinal, superior-inferior), Y-axis (transverse, left-right) and Z-axis (vertical, posterior-anterior) via stepper motors (resolution = 0.007 mm, www.stepperonline.com, China) (Fig. 3). A string potentiometer (resolution = 0.020 mm, accuracy ±0.010 mm, TE Connectivity, USA) is used to record Z-axis displacement. A vertically-oriented laser assists the operator to align the RIH upon pre-determined anatomical landmarks (GLX Laser Site, Barska). During spinal stiffness assessment, the VerteTrack applies discrete loads via addition of weighted plates (“plates”) with a nominal mass of 1 kg each (RIH + *k* plates; *k* = 0, 6). These loads were selected as they represent loads that have previously been used in VerteTrack studies [22, 23, 26] and are comparable to loads applied in other mechanical indentation studies [14, 20]. Plates were numbered and always added in the same order for each indentation cycle. For more detail about the VerteTrack see Brown et al. 2017 [22].

### Methods of indentation

The VerteTrack can perform two modes of indentation testing: single-level and multiple-level continuous indentation. Single-level indentation assesses a single spinal level and requires the operator to position the RIH directly above the target tissue. Loads are then applied incrementally to the spine in a posterior to anterior direction with the resulting deformation of the spinal tissues recorded (Z-axis displacement). Multiple-level continuous indentation requires the operator to first identify the spinal trajectory that the RIH will travel within the horizontal (X-Y) plane. This is achieved by manually aligning each spinous process (determined by palpation or ultrasonography) with the RIH using the embedded laser pointer. The laser points are memorised by the device and then replayed to move the RIH continuously along the same pre-defined trajectory for each successive load. The resolution of the RIH is identical to the resolution of stepper motors (0.007 mm).

### Load and displacement precision

**Fig. 4:**
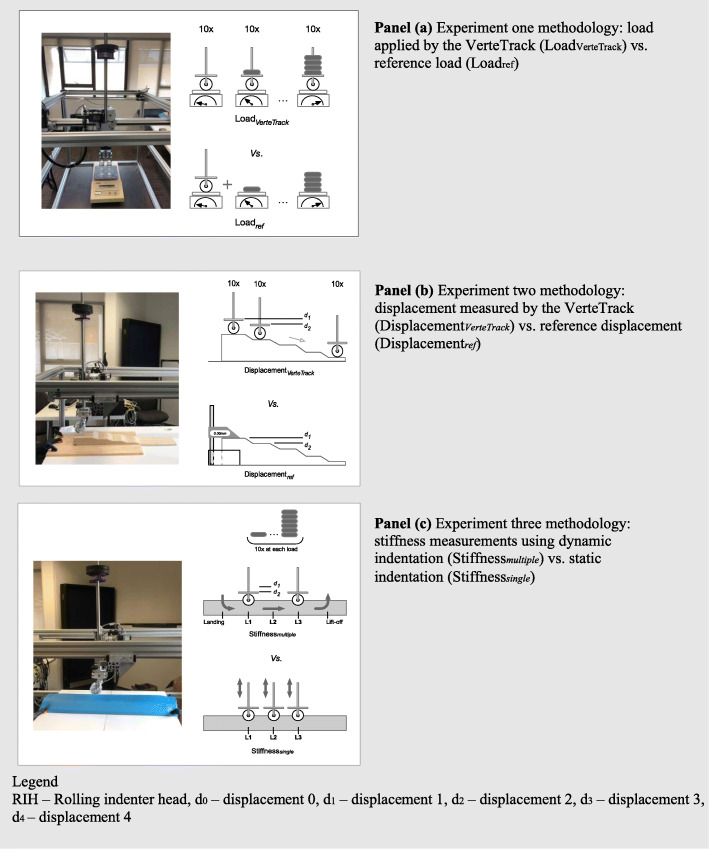
**a** Experiment one methodology: load applied by the VerteTrack (Load_VerteTrack_) vs. reference load (Load_ref_). **b** Experiment two methodology: displacement measured by the VerteTrack (Displacement_*VerteTrack*_) vs. reference displacement (Displacement_*ref*_). **c** Experiment three methodology: stiffness measurements using multiple-level continuous indentation (Stiffness_*multiple*_) vs. single-level indentation (Stiffness_*single*_)**.** Legend: RIH – Rolling indenter head, d_0_ – displacement 0, d_1_ – displacement 1, d_2_ – displacement 2, d_3_ – displacement 3, d_4_ – displacement 4

Load precision (random error) of the VerteTrack was estimated by the coefficient of variation (CV = SD / load mean) over 10 repetitions for each load. The RIH was measured using recently calibrated digital scales (OHAUS, model TS4KD: Resolution 0.1 g, accuracy ±0.07 g) (Fig. 4, panel a). Each plate was added to the RIH, then repeated up to a total of 5 plates. Loads were converted to Newtons (N) using mass (kg) x gravity (9.81 m/s^2^). Displacement precision (z-axis, depth) of the VerteTrack was also estimated using coefficient of variation over 10 repetitions at each of 6 discrete levels of the RIH on a custom-engineered wooden wedge to simulate tracking of a spinal sagittal curve (Fig. 4, panel b).

### Load and displacement bias

Load bias (systematic error) was estimated by comparing each load delivered through the VerteTrack against the same load externally. Mean load bias was estimated by calculating the differences between reference loads and loads measured by the VerteTrack, and the 95% confidence interval of the difference [25]. Reference loads were calculated by the addition of successive plates placed directly upon the digital scale (i.e. not through the VerteTrack RIH) plus the load measured through the RIH alone. Each reference load (*k* plates; *k* = 1, 5) was measured ten times. Displacement bias was also estimated using the same method employed to determine load bias. Mean displacement bias was determined over 10 repetitions at each of 6 discrete levels as reported by the VerteTrack, compared to an external digital calliper (Wixey, WR200: Resolution = 0.05 mm, accuracy ±0.025 mm) (Fig. 4, panel b).

#### Comparison of single-level and multiple-level continuous operation

A method-comparison experiment was conducted to evaluate the performance of VerteTrack for measurement of stiffness during multiple-level continuous and single-level (reference) modes of operation. Terminal stiffness values (i.e. the ratio of the maximum load to the maximum displacement) [26] were used in our analysis. The stiffness of a deformable foam test medium (AIREX® balance beam, Switzerland) was measured during both single-level and multiple-level continuous modes of operation to simulate measurement at a single vertebral level and across multiple vertebral levels respectively. The test medium was chosen to emulate the physiological stiffness encountered for the in vivo adult lumbar spine (range: 2–10 N/mm) [12, 20, 26]. Five equidistant locations (5 cm apart) were marked on the foam medium along a straight line (RIH landing, L1, L2, L3 and RIH lift-off) for stiffness assessment (Fig. 4, panel c).

### Precision during single-level and multiple-level continuous indentation

Precision of the VerteTrack during measurement of stiffness on the test medium was estimated by the coefficient of variation (CV = SD / stiffness mean) over 300 trials for both single-level and multiple-level continuous indentation. Stiffness was measured during multiple-level continuous indentation (Stiffness_*multiple*_) and single-level indentation (Stiffness_*single*_) at three discrete locations (L1, L2, L3) on the medium. Incremental loads (plates) were added to the RIH in a predefined sequence (RIH + *k*; *k* = 1, 6). Between each trial, 90 s elapsed to allow for any residual deformation to resolve. Between each cycle (six trials of increasing load), an additional 5 min elapsed to allow any residual deformation to resolve after the maximum load was applied to the medium. A total of ten cycles were performed.

### Single-level versus multiple-level continuous indentation

Each trial for Stiffness_*multiple*_ was compared to Stiffness_*single*_, to quantify bias between indentation methodologies we calculated the stiffness differences and 95% confidence intervals of the difference. Bias calculation, and a plot of raw stiffness data were conducted to assist interpretation. In addition, Lin’s Concordance Correlation Coefficient (LinCCC, Rc) was reported for load and displacement. LinCCC tests both agreement and linearity [27]. The strength of agreement was graded as “almost perfect” (Rc > 0.99), “substantial” (Rc > 0.95–0.99), “moderate” (Rc > 0.90–0.95), or “poor” (Rc < 0.90) [28]. Alpha was set at 0.05 for all statistical significance tests of agreement.

## Results

### Load and displacement precision

Six measures relating to RIH loading (Load_VerteTrack_) were obtained beginning with no load 16.557 N (95%CI: 16.470 to 16.645, RIH only) then increasing by the addition of single masses. The coefficient of variation (CV) ranged from 0.03 to 0.27% depending upon the applied load (Table 1).

**Table 1:** Precision of VerteTrack applied load

Indenter head loading	Load_*VerteTrack*_^a^ (N)	95%CI (N)	SD	CV
RIH only	16.557	16.470 to 16.645	0.045	0.27%
RIH + 1 plate	27.757	27.701 to 27.814	0.029	0.10%
RIH + 2 plates	38.662	38.589 to 38.735	0.037	0.10%
RIH + 3 plates	49.583	49.479 to 49.688	0.053	0.11%
RIH + 4 plates	60.687	60.592 to 60.783	0.049	0.08%
RIH + 5 plates	71.461	71.420 to 71.502	0.021	0.03%

*CV* Coefficient of variation, *RIH* Rolling indenter head, *SD* standard deviation

^a^Average of 10 measurements at each load. All loads measured with digital scale (OHAUS, model TS4KD: Resolution = 0.1 g, accuracy ±0.07 g. Equivalent to resolution = 0.001 N, accuracy ±0.0007 N)

Six discrete RIH displacements were then measured (Displacement_*VerteTrack*_) beginning at a baseline value of 60.03 mm (95%CI: 60.01 to 60.05 mm, highest level) then increasing to 12.08 mm (95%CI: 12.00 to 12.16 mm, lowest level). The CV ranged from 0 to 0.32% depending upon the level of the wedge (Table 2).

**Table 2:** Precision of the VerteTrack RIH displacement

Wedge level	RIH displacement relative to table-top^a^ (mm)	95%CI (mm)	SD	CV
d_0_ (landing point)	60.03	60.01 to 60.05	0.04	0.01%
d_1_ (highest level)	60.02	60.02 to 60.02	0.01	0.00%
d_2_	48.30	48.29 to 48.31	0.01	0.00%
d_3_	36.13	36.12 to 36.13	0.01	0.01%
d_4_	23.82	23.82 to 23.83	0.01	0.01%
d_5_ (lowest level)	12.08	12.00 to 12.16	0.14	0.32%

*CV* Coefficient of variation, *RIH* Rolling indenter head, *SD* standard deviation

^a^Average of 10 measurements at each displacement. All displacements were measured by the string potentiometer relative to the table-top (TE Connectivity, USA, Resolution = 0.020 mm, accuracy ±0.010 mm)

### Load and displacement bias

The calculated reference loads (Load_*ref*_) ranged from 27.757 N (95%CI: 27.701 to 27.814 N, RIH + 1 plate) to 71.461 N (95%CI: 71.420 to 71.502 N, RIH + 5 plates). There was a statistically significant (*p* < .001) systematic mean bias for the VerteTrack load (Load_*VerteTrack*_), compared to the reference load (Load_*ref*_) of − 0.123 N (95%CI: − 0.182 to 0.428 N, p < .001) (supplementary figure 1, panel a). Lin’s Concordance Correlation Coefficient showed almost perfect agreement (Rc = 1.0, 95% CI: 1.0 to 1.0) between Load_*VerteTrack*_ and Load_*ref*_ (supplementary figure 2, panel a).

The reference displacement (Displacement_*ref*_) as measured by the digital calliper ranged from 12.03 mm (95%CI: 11.98 to 12.08 mm) to 60.08 mm (95%CI: 60.02 to 60.13 mm). There was no statistically significant (*p* = .001) systematic bias for the VerteTrack displacement (Displacement_*VerteTrack*_) as compared to the reference displacement (Displacement_*ref*_) (mean difference = 0.02 mm, 95%CI: − 0.09 to 0.14 mm, *p* < .001) (supplementary figure 1, panel b). Lin’s Concordance Correlation Coefficient demonstrated an almost perfect agreement (Rc = 1.0, 95% CI: 1.0 to 1.0) between Displacement_*VerteTrack*_ and Displacement_*ref*_ (supplementary figure 2, panel b).

#### Stiffness measurements

##### Performance at different loads

**Fig. 5:**
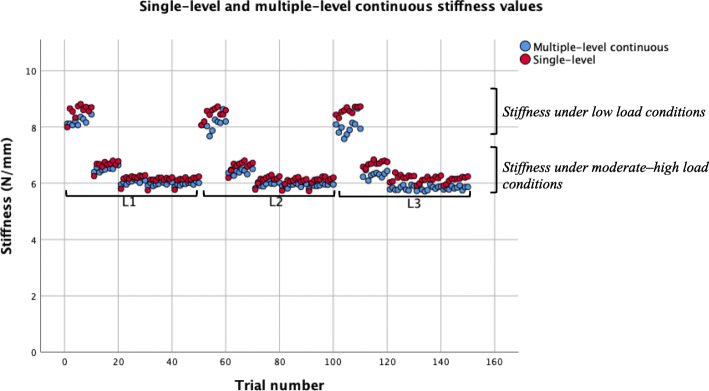
Multiple-level continuous and single-level stiffness data across three locations (L1, L2 and L3) on the AIREX balance beam. L1: trial 1–50, L2: trial 51–100, L3: trial 101–150. Histogram for each location

For the purposes of this study, we classified low load conditions as 27.757 N and 38.662 N (1 and 2 plates), and moderate to high loads as 49.583 N, 60.687 N and 71.461 N (3–5 plates). The terminal stiffness values for low load conditions ranged from 6.09 to 8.81 N/mm, and moderate-high load conditions yielded stiffness values ranging from 5.70 to 6.38 N/mm. Under low load conditions, the terminal stiffness grand mean was 7.43 N/mm. As for moderate to high load conditions, the terminal stiffness grand mean was 6.03 N/mm (Fig. 5). Figure 5 shows a graphical representation of the effects of low versus moderate-high loading on stiffness values.

##### Performance of single-level versus multiple-level continuous indentation modes

To determine the precision of stiffness measured by the VerteTrack (Stiffness_*multiple*_ and Stiffness_*single*_) at three locations on the AIREX balance beam, CV was calculated for each load (Table 3). The CV at each load for Stiffness_*single*_ ranged from 2.0 to 2.3% and Stiffness_*multiple*_ ranged from 1.4 to 3.2%.

**Table 3:** Coefficient of variation for Stiffness_*multiple*_ and Stiffness_*single*_

VerteTrack mode	Coefficient of variation (CV) at each load					
	RIH + 1	RIH + 2	RIH + 3	RIH + 4	RIH + 5	Mean
Single-level	2.3%	2.2%	2.1%	2.0%	2.1%	**2.2%**
Multiple-level continuous	3.2%	2.2%	2.2%	1.7%	1.4%	**2.2%**

*RIH* Rolling indenter head

Multiple-level continuous indentation (Stiffness_*multiple*_) and single-level indentation (Stiffness_*single*_) were compared at three discrete locations (L1, L2 and L3) on the AIREX balance beam. There was a negative systematic bias for Stiffness_*multiple*_, compared to Stiffness_*single*_ of − 0.25 N/mm (95%CI − 0.67 to 0.17, *p* < 0.001) (supplementary figure 1, panel c).

## Discussion

This is the first mechanical spinal stiffness device to be evaluated for the bench-top performance (accuracy), which is essential to establish internal validity of the VerteTrack. Both single-level and multiple-level continuous indentation modes demonstrated high levels of precision and agreement, despite a small negative systematic bias for multiple-level continuous compared to single-level indentation (− 0.25 N/mm, equivalent to 4% lower stiffness). It is unclear if this difference is clinically relevant as there is currently no published data to support a minimal clinically important difference (MCID) for the assessment of spinal stiffness, nor standards for different indentation modes [3, 11]. More broadly, mechanical devices must first be used to collect baseline spinal stiffness data in a human population in order to determine a MCID, while on the other hand, an MCID cannot be calculated without understanding the performance of a measurement as is described here. Such baseline data will allow for more robust conclusions regarding differences between single-level and multiple-level continuous indentation.

The plot of raw single-level and multiple-level continuous stiffness values demonstrated that higher stiffness values were obtained under low loads compared with moderate-high loads where stiffness remained at approximately 6 N/mm (Fig. 5). This is likely attributed to properties of the viscoelastic foam medium. In vivo testing observed the inverse relationship between load and stiffness, that is a positive relationship between load and stiffness [23]. It would be advantageous to identify on a human population a specific load that yeilds the most useful spinal stiffness information. As reducing the number of loads would further reduce assessment time. In addition, results from in vivo testing suggest that the device provided reliable stiffness values, irrespective of load [23].

### Limitations

This study was performed on a viscoelastic foam medium, without the presence of physiological properties known to influence spinal stiffness (such as breathing, spinal extensor muscle contraction and abdominal muscle contraction) [3, 11]. Also, it is unclear to what extent the observed phenomena can be attributed to the medium and whether a human population would emulate similar findings. To quantify bias, the level of agreement between multiple-level continuous stiffness measurements was compared to a reference standard. Single-level indentation was used as a proxy reference standard, given that it is the more established method of indentation reported in the literature. Unfortunately, there is no ‘gold standard’ to ascertain spinal stiffness in human participants.

### Clinical utility of mechanical measurement of spinal stiffness

It is unclear whether the use of mechanical spinal stiffness measurement devices in a clinical setting would aid in the patient’s diagnosis, prognosis, treatment, or clinical outcomes. There are inconsistencies in the literature regarding the relationship between pain, disability and spinal stiffness, however emerging research into the sub-grouping of patients into responders and non-responders to spinal manipulative therapy have yielded promising results [16]. Mechanical spinal stiffness devices currently have limited utility in clinical practice until further research can identify specific populations that may benefit from the assessment procedure.

## Conclusion

The Vertetrack demonstrated good bench-top performance through high precision, linearity, and low systematic bias compared to reference values. When combined with recently published clinical reliability data, the VerteTrack demonstrates high levels of internal validity. The novel multiple-level continuous indentation mode offers potential for increased time efficiency in future clinical trials without compromise in stiffness measurement or patient comfort.

## Supplementary information

**Additional file 1: Figure S1. Panel a)** The Bland-Altman plot demonstrates a statistically significant bias (*p* < .001) for loads delivered by the VerteTrack compared to the calibration sample (− 0.123 N; 95%CI − 0.182 to 0.428 N, *p* < .001). Open circles (50 data points) represent the magnitude of bias (N) = Load_ref_ - Load_VerteTrack_. **Panel b) The** Bland-Altman plot demonstrates no statistically significant bias (*p* = .001) for displacement as measured by the VerteTrack compared to a digital calliper (+ 0.02 mm, 95% CI − 0.09 to 0.14 mm, *p* < .001). Open circles (60 data points) represent the magnitude of bias (mm) = Displacement_*ref*_ - Displacement_*VerteTrack.*_**Panel c) The** Bland-Altman plot demonstrates a statistically significant (*p* < .001) negative bias for multiple-level continuous vs. single-level stiffness, of 0.25 N/mm (95%CI − 0.67 to 0.17 N/mm, *p* < 0.001). Open circles (150 data points) represent the magnitude of bias (N) = Stiffness_*multiple*_ - Stiffness_*single.*_ Legend: RIH – Rolling indenter head, d_0_ – displacement 0, d_1_ – displacement 1, d_2_ – displacement 2, d_3_ – displacement 3, d_4_ – displacement 4. **Figure S2. Panel a)** Lin’s Concordance Correlation Coefficient for VerteTrack load vs. the reference sample to demonstrate almost perfect agreement (Rc = 1.0, 95% CI 1.0 to 1.0). Open circles (50 data points) represent co-ordinates (Load_ref_, Load_VerteTrack_) at loads (RIH + k plates; k = 1, 5). **Panel b)** Lin’s Concordance Correlation Coefficient for VerteTrack displacement vs. the digital calliper demonstrated an almost perfect agreement (Rc = 1.0, 95% CI 1.0 to 1.0). Open circles (60 data points) represent co-ordinates (Displacement_ref_, Displacement_VerteTrack_) for each wedge level (d_0_–d_5_)**.** Legend: RIH – Rolling indenter head, d_0_ – displacement 0, d_1_ – displacement 1, d_2_ – displacement 2, d_3_ – displacement 3, d_4_ – displacement 4.

## Data Availability

The full data set used for the analysis is available on request from the corresponding author.

## Abbreviations

MSSA: Manual spinal stiffness assessment
RIH: Rolling indenter head
ICC: Intraclass correlation coefficient
MCID: Minimally clinically important difference
CV: Coefficient of variation
CI: Confidence interval
N: Newtons
L1: Location 1
L2: Location 2
L3: Location 3
d_0_: Displacement 0
d_1_: Displacement 1
d_2_: Displacement 2
d_3_: Displacement 3
d_4_: Displacement 4
