# Supplementary material for: The bench-top accuracy of the VerteTrack spinal stiffness assessment device
Source: Chiropr Man Therap. 2020 Aug 18;28:42. doi: 10.1186/s12998-020-00331-8 (PMC7433107; doi:10.1186/s12998-020-00331-8)
Supplement: Supplementary file 1 — Additional file 1: Figure S1. Panel a) The Bland-Altman plot demonstrates a statistically significant bias (p < .001) for loads delivered by the VerteTrack compared to the calibration sample (− 0.123 N; 95%CI − 0.182 to 0.428 N, p < .001). Open circles (50 data points) represent the magnitude of bias (N) = Loadref - LoadVerteTrack. Panel b) The Bland-Altman plot demonstrates no statistically significant bias (p = .001) for displacement as measured by the VerteTrack compared to a digital calliper (+ 0.02 mm, 95% CI − 0.09 to 0.14 mm, p < .001). Open circles (60 data points) represent the magnitude of bias (mm) = Displacementref - DisplacementVerteTrack.Panel c) The Bland-Altman plot demonstrates a statistically significant (p < .001) negative bias for multiple-level continuous vs. single-level stiffness, of 0.25 N/mm (95%CI − 0.67 to 0.17 N/mm, p < 0.001). Open circles (150 data points) represent the magnitude of bias (N) = Stiffnessmultiple - Stiffnesssingle. Legend: RIH – Rolling indenter head, d0 – displacement 0, d1 – displacement 1, d2 – displacement 2, d3 – displacement 3, d4 – displacement 4. Figure S2. Panel a) Lin’s Concordance Correlation Coefficient for VerteTrack load vs. the reference sample to demonstrate almost perfect agreement (Rc = 1.0, 95% CI 1.0 to 1.0). Open circles (50 data points) represent co-ordinates (Loadref, LoadVerteTrack) at loads (RIH + k plates; k = 1, 5). Panel b) Lin’s Concordance Correlation Coefficient for VerteTrack displacement vs. the digital calliper demonstrated an almost perfect agreement (Rc = 1.0, 95% CI 1.0 to 1.0). Open circles (60 data points) represent co-ordinates (Displacementref, DisplacementVerteTrack) for each wedge level (d0–d5). Legend: RIH – Rolling indenter head, d0 – displacement 0, d1 – displacement 1, d2 – displacement 2, d3 – displacement 3, d4 – displacement 4. [file 12998_2020_331_MOESM1_ESM.docx]

#### **Supplementary figure 1**


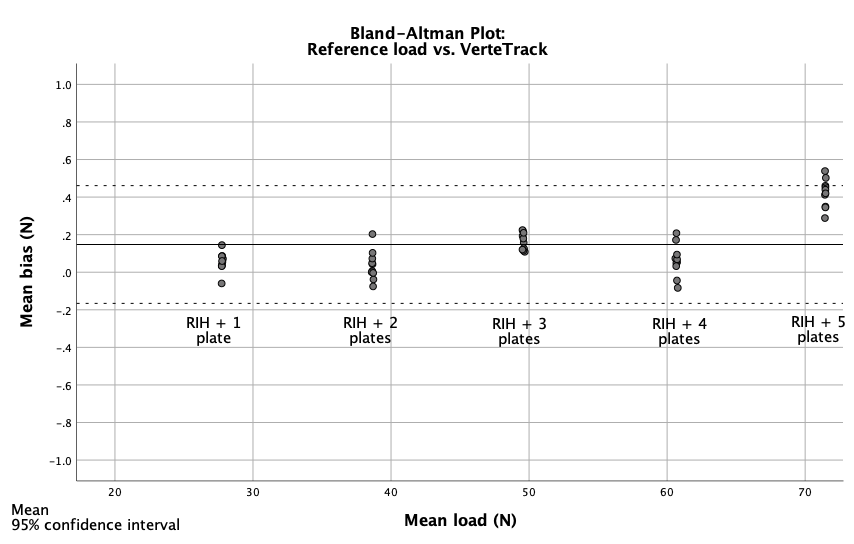


**Panel (a)** Bland-Altman plot to demonstrate a statistically significant bias (p < .001) for loads delivered by the VerteTrack compared to the calibration sample (-0.123N; 95%CI -0.182 to 0.428, p < .001). Open circles (50 data points) represent the magnitude of bias (N) = Load_ref_ - Load_VerteTrack_


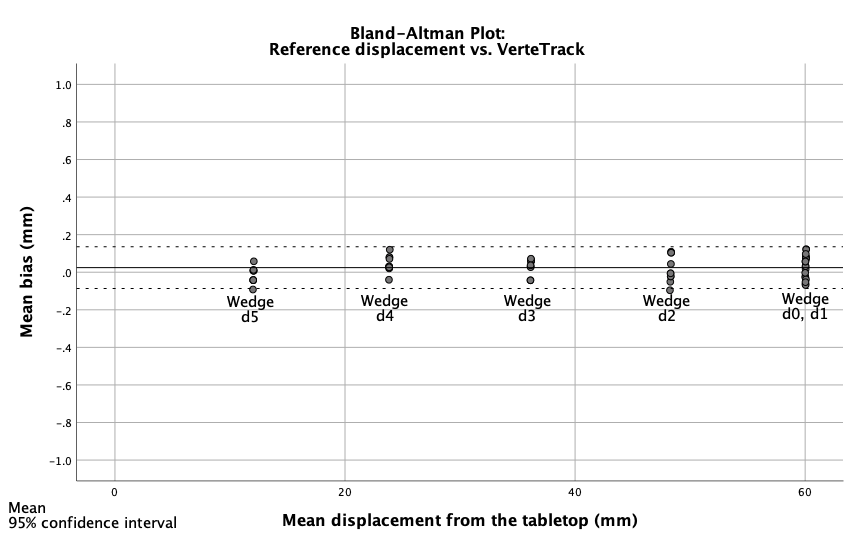


**Panel (b)** Bland-Altman plot to demonstrate no statistically significant bias (p = .001) for displacement as measured by the VerteTrack compared to the digital calliper (+0.02mm, 95% CI -0.09 to 0.14, p < .001). Open circles (60 data points) represent the magnitude of bias (mm) = Displacement*_ref_*  - Displacement*_VerteTrack_*


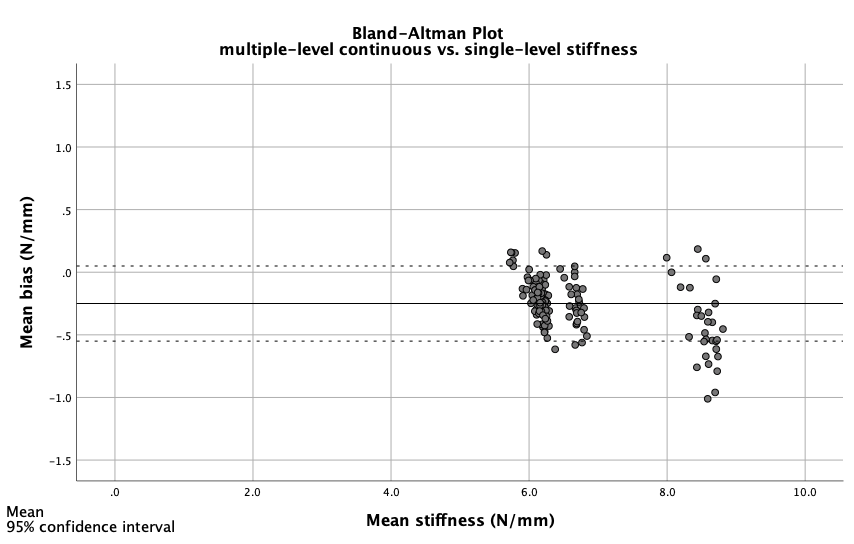


Legend

RIH – Rolling indenter head, d_0_ – displacement 0, d_1_ – displacement 1, d_2_ – displacement 2, d_3_ – displacement 3, d_4_ – displacement 4

**Panel (c)** Bland-Altman plot demonstrating the negative bias for multiple-level continuous vs. single-level stiffness. Bland-Altmann plot to demonstrate a statistically significant (p < .001) bias for stiffness calculated using multiple-level continuous indentation, compared to stiffness calculated using single-level indentation of -0.25N/mm (95%CI -0.67 to 0.17, p < .001). Open circles (150 data points) represent the magnitude of bias (N) = Stiffness*_multiple_* - Stiffness*_single_*

*Stiffness under
moderate–high load conditions*

*Stiffness under
low load conditions*


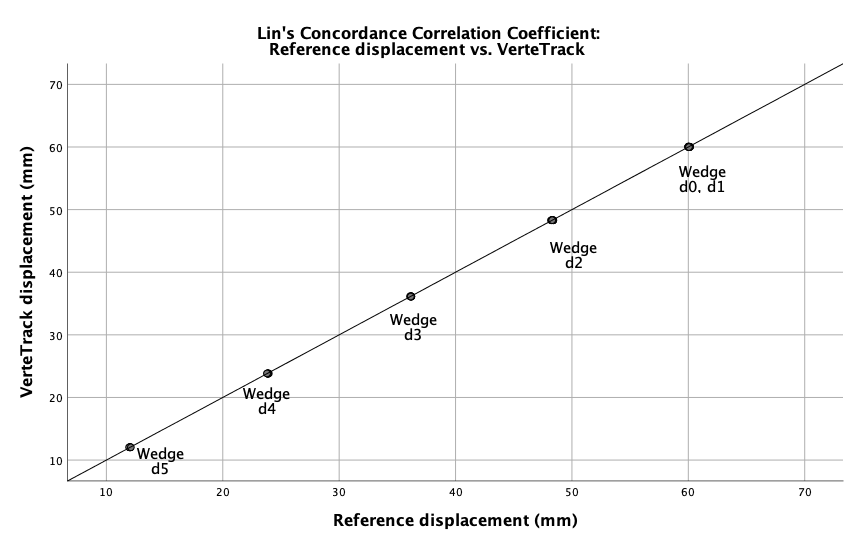

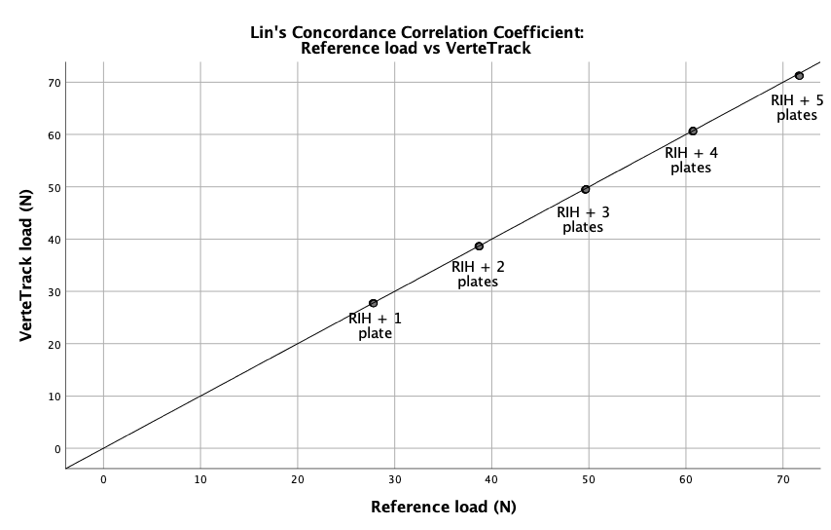


**Supplementary figure 2**

Legend

RIH – Rolling indenter head, d_0_ – displacement 0, d_1_ – displacement 1, d_2_ – displacement 2, d_3_ – displacement 3, d_4_ – displacement 4

**Panel (a)** Lin’s Concordance Correlation Coefficient for VerteTrack load vs. the reference sample to demonstrate almost perfect agreement (Rc = 1.0, 95% CI 1.0 to 1.0). Open circles (50 data points) represent co-ordinates (Load_ref_, Load_VerteTrack_) at loads (RIH + k plates; k=1, 5)

**Panel (b)** Lin’s Concordance Correlation Coefficient for VerteTrack displacement vs. the digital calliper demonstrated an almost perfect agreement (Rc = 1.0, 95% CI 1.0 to 1.0). Open circles (60 data points) represent co-ordinates (Displacement_ref_, Displacement_VerteTrack_) for each wedge level (d_0_–d_5_)
